# Supplementary material for: Capillary compression induced outstanding n-type thermoelectric power factor in CNT films towards intelligent temperature controller
Source: Nat Commun. 2024 Jul 4;15:5617. doi: 10.1038/s41467-024-50057-x (PMC11224367; doi:10.1038/s41467-024-50057-x)
Supplement: Supplementary file 1 — Supplementary Information [file 41467_2024_50057_MOESM1_ESM.pdf]

Supporting information

Capillary compression induced outstanding n-type thermoelectric power factor in CNT films towards intelligent temperature controller

Hong Wang[1,2*‡], Kuncai Li [1‡], Xin Hao[1,2], Jiahao Pan[1], Tiantian Zhuang[1], Xu Dai[1], Jing Wang[1,2], Bin Chen[1,2], Daotong Chong[1,2]

[1] State Key Laboratory of Multiphase Flow in Power Engineering & Frontier Institute of Science and Technology, Xi’an Jiaotong University, Xi’an, 710054, China

[2] School of Energy and Power Engineering, Xi’an Jiaotong University, Xi’an, 710054, China

E-mail: hong.wang@xjtu.edu.cn

‡These two authors contribute equally to the paper.

Calculation of the weighted mobility

The weighted mobility (μ_w) was estimated from the electrical conductivity and thermopower, which reflects the carrier mobility weighted by the density of states as follows:

$$\mu_w = \frac{3h^3\sigma}{8\pi e(2m_e k_B T)^{3/2}}\left[\frac{exp\left[\frac{|S|}{k_B/e}-2\right]}{1+exp\left[-5\left(\frac{|S|}{k_B/e}-1\right)\right]}+\frac{\frac{3}{\pi^2}\frac{|S|}{k_B/e}}{1+exp\left[5\left(\frac{|S|}{k_B/e}-1\right)\right]}\right]$$

Where m_e, k_B, *h, e*, σ, and S are the electron mass, Boltzmann constant, Planck constant, electronic charge, electrical conductivity, and Seebeck coefficient, respectively. The weighted mobility gives nearly the same information about charge carrier mobility as the Hall mobility and thus it has been widely used to investigate charge carrier transport mechanisms in previous literatures.[1, 2, 3, 4]

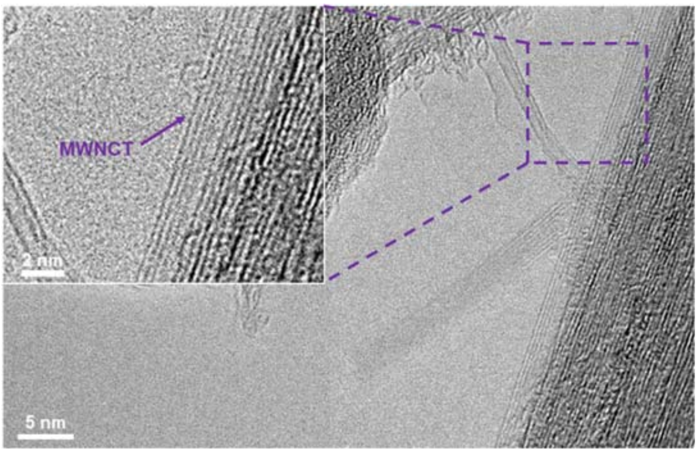

Figure S1. The TEM images of CNT.

High-resolution transmission electron microscopy (TEM) images showed that the majority of the CNTs were multi-walled carbon nanotubes (MWCNTs). Similar results have been reported in previous works that MWCNTs were obtained when the same method was used in the synthesis process.[5] As it is known that the band gap is inversely proportional to the diameter of CNTs, MWCNTs typically exhibit metal-like electrical properties.[6, 7] The high Seebeck coefficient of the CNT films can be attributed to the Fermi energy level being close to the 1D van Hove singularity of CNTs as suggested in the previous literature.[6, 7, 8, 9, 10]

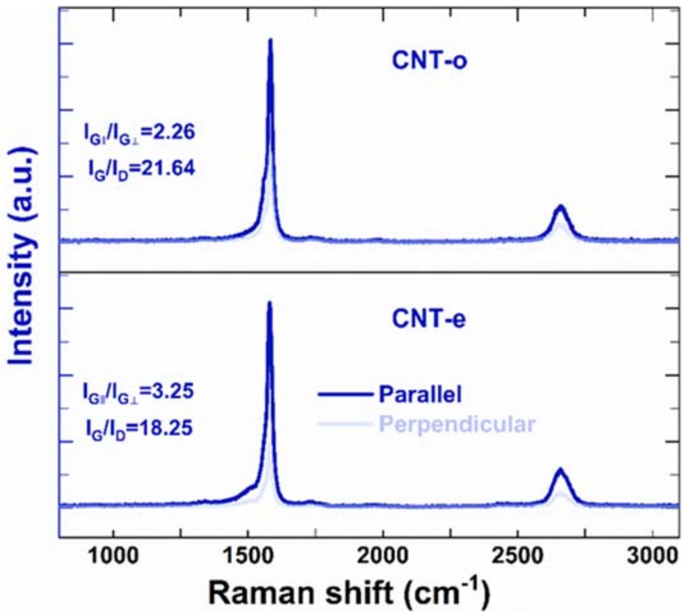

Figure S2. The Raman spectra of CNT-o films and CNT-e films.

CNT films were proved to be anisotropic by polarized Raman spectroscopy as well, as shown in Figure S2. The G-band intensity of CNT-e films in the direction parallel to the rolling direction ($I_{G//}$) is about 3.25 times higher than that in the perpendicular direction ($I_{G\perp}$), while the $I_{G//}$: $I_{G\perp}$ in CNT-o films is only 2.26. The higher $I_{G//}$: $I_{G\perp}$ value indicates the better alignment of CNTs in CNT-e films.

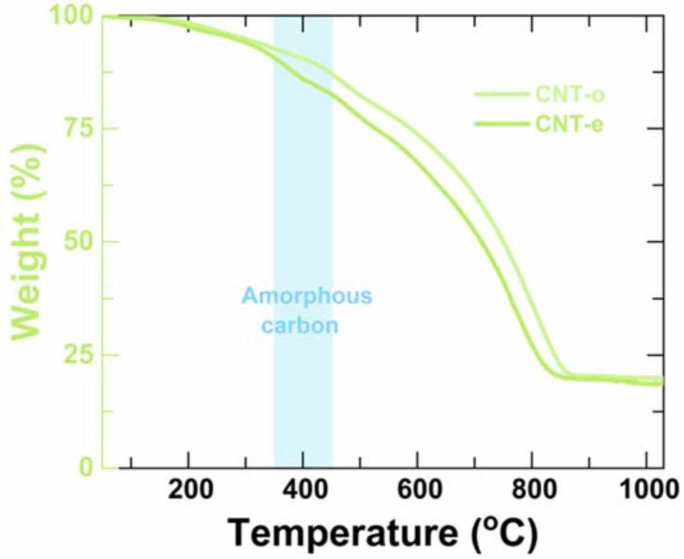

Figure S3. The TGA curve of CNT-o films and CNT-e films.

CNT-o films and CNT-e films showed similar change trends and residual masses, indicating that ethanol treatment did not have a significant effect on CNT quality. The amorphous carbon content of CNT-o and CNT-e films was roughly calculated to be 5.5% and 8.1%, respectively, based on the weight change rate within 350-450 °C, as shown in Figure S3.[5, 11, 12, 13] This is also consistent with the results of the Raman spectra shown in Figure S2 that I_G/I_D of CNT-e is slightly lower than that of CNT-o.

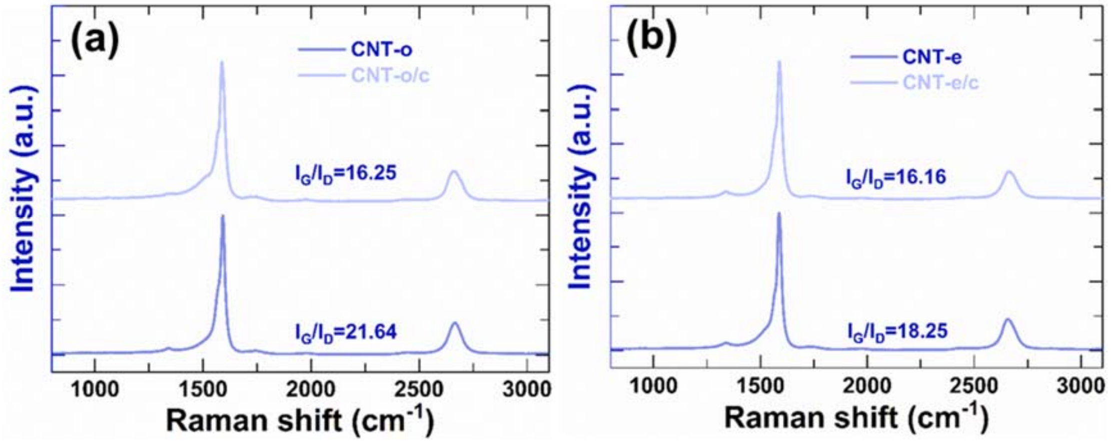

Figure S4. The Raman spectra of CNT-o films, CNT-e films, CNT-o/c films and CNT-e/c films.

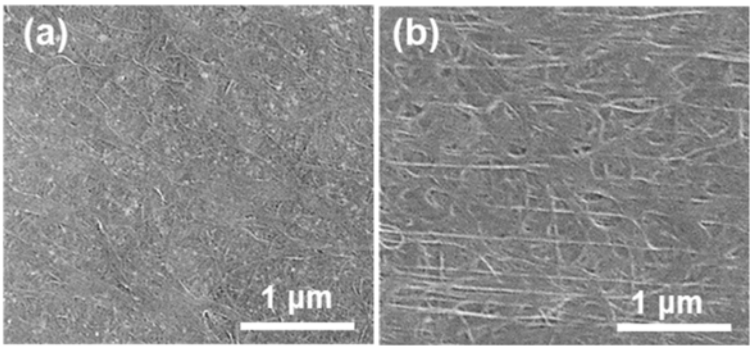

Figure S5. SEM images of CNT-o/c films and CNT-e/c films.

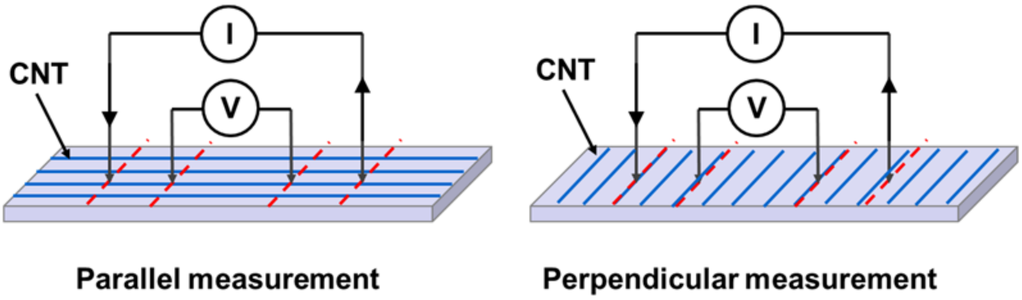

Figure S6. The schematic of the anisotropic electrical conductivity measurements for CNT films.

The electrical conductivity of the film was measured by commercial equipment (NETZSCH, SBA-458, Germany) with a four-probe method. All measurements were carried out at room temperature under argon protection and the CNT film samples were cut into strips 20 mm long and 4 mm wide. The electrical conductivity (σ) of the samples was calculated by the equation: $\sigma = 1/\rho = L/RA$, where ρ was the resistivity, *L* was the length of the sample between the electrodes, *R* was the resistance and *A* was the cross-sectional area of the sample. This method has been widely used for the electrical conductivity measurement of films.[5, 7, 9, 10, 14, 15, 16, 17]

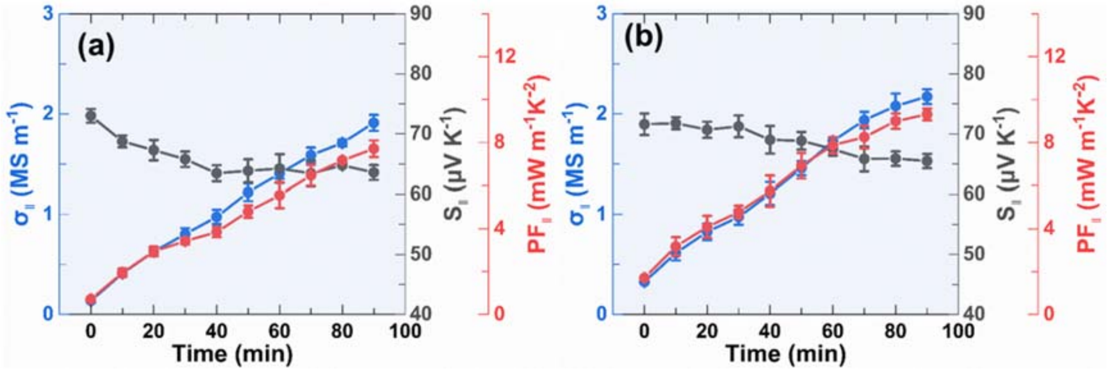

Figure S7. The TE properties in the parallel direction of CNT-o films (a) and CNT-e (b) films as a function of compressing time.

To understand the change of the electrical conductivity and the Seebeck coefficient of the CNT films during compressing, it would be better to focus on the "effective" conducting components which are CNT in the films. It is believed that the change in carrier mobility is responsible for the high power factor rather than the density of states.

The increase of the electrical conductivity of CNT films was due to the decrease of the porosity. SEM images in Figure S5 indicated that the CNT films became dense after being compressed. The packing density of CNTs in the films before compressing was lower than that of the CNTs in the films after compressing (Figure 1a, 1b and Figure S5), which subsequently resulted in lower electrical conductivity of the CNT films before compressing than that of the CNT films after compressing (Figure 1c). The electrical conductivity changes of the CNT films before and after compressing could be well understood with Maxwell-Eucken's equation as shown below:

$$\sigma_P = \sigma_0 \times \frac{1-P}{1+\beta P} \quad (1)$$

where P is the porosity, σ_P is the total electrical conductivity of a porous material, σ_0 is the intrinsic electrical conductivity of the materials, and β is the constant number determined by the conditions of the pores. The value of β is between 1.0 and 3.0 when the shape of the pores is almost the spherical style.[18, 19, 20] When the CNT films were compressed, the porosity decreased (the packing density increased). The well alignment and dense packing of CNT

resulted in the improvement of the carrier mobility in the films. Therefore, the electrical conductivity of the compressed CNT films was higher than that of the CNT films before compressing. Similar results have also been reported in previous works that the electrical conductivity is inversely proportional to the thickness (proportional to the density) of CNT films or their composite films after compressing.[21, 22, 23, 24, 25, 26] In the meanwhile, the compressing process was a physical process, which would not change the chemical environment of a single CNT. Ultraviolet Photoelectron Spectroscopy (UPS) was performed with gold as a reference to identify the work function change of CNT-e and CNT-e/c films (Figure S9). The obtained work function of CNT-e film and CNT-e/c film exhibited similar values, indicating that the chemical environment of the CNT remained constant (the Fermi energy level is unchanged) before and after compressing. In addition, the pressure used in this work was only ~100 MPa which was far behind the pressure required to change the cylinder structure of a single CNT (serval GPa).[27, 28, 29] Therefore, the Seebeck coefficient of the CNT films changed little after compressing.[9, 10, 16, 17] The increased electrical conductivity and maintained Seebeck coefficient resulted in the increase of thermoelectric power factor of CNT films after compressing in Figure 2d in the original manuscript.

The energy-filtering effect usually emerges from the barrier blockage of low-energy carriers by the potential barrier, resulting in a significant increase in the Seebeck coefficient, while the electrical conductivity remains constant.[30, 31] The energy filtering effect was usually observed in carbon nanotube-organic/inorganic composites, as reported in previous literature.[32, 33] However, this effect may not fulfill the scenario of CNT only films in this work since the Seebeck coefficient of the CNT films maintained nearly constant before and after compressing (Figure 2c).

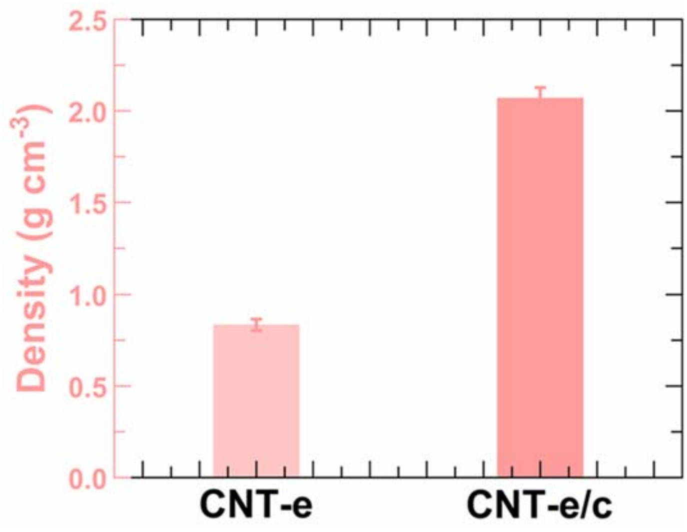

Figure S8. The density of the CNT-e films before and after compressing.

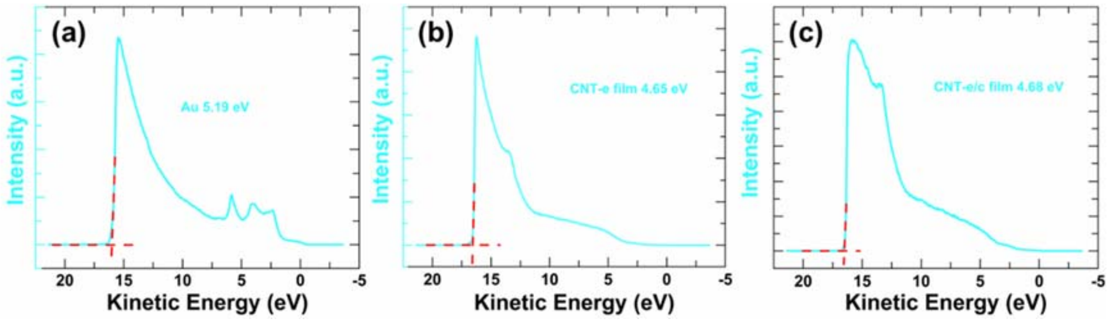

Figure S9. UPS results of CNT-a and CNT-e/c films.

The work function was obtained by the equation: $\phi = h\nu + |E_F| - |E_{cutoff}|$, where ϕ was the work function, $h\nu$ was the incoming photon energy from the He I source of 21.2 eV, and $|E_F| - |E_{cutoff}|$ was the difference in energy between the onset of the secondary electrons and the Fermi edge.[6, 7] The obtained work function of CNT-e film and CNT-e/c film exhibited similar values, indicating that the chemical environment of the CNT remained constant (the Fermi energy level is unchanged) before and after compressing, which resulted in the maintenance of the Seebeck coefficient.

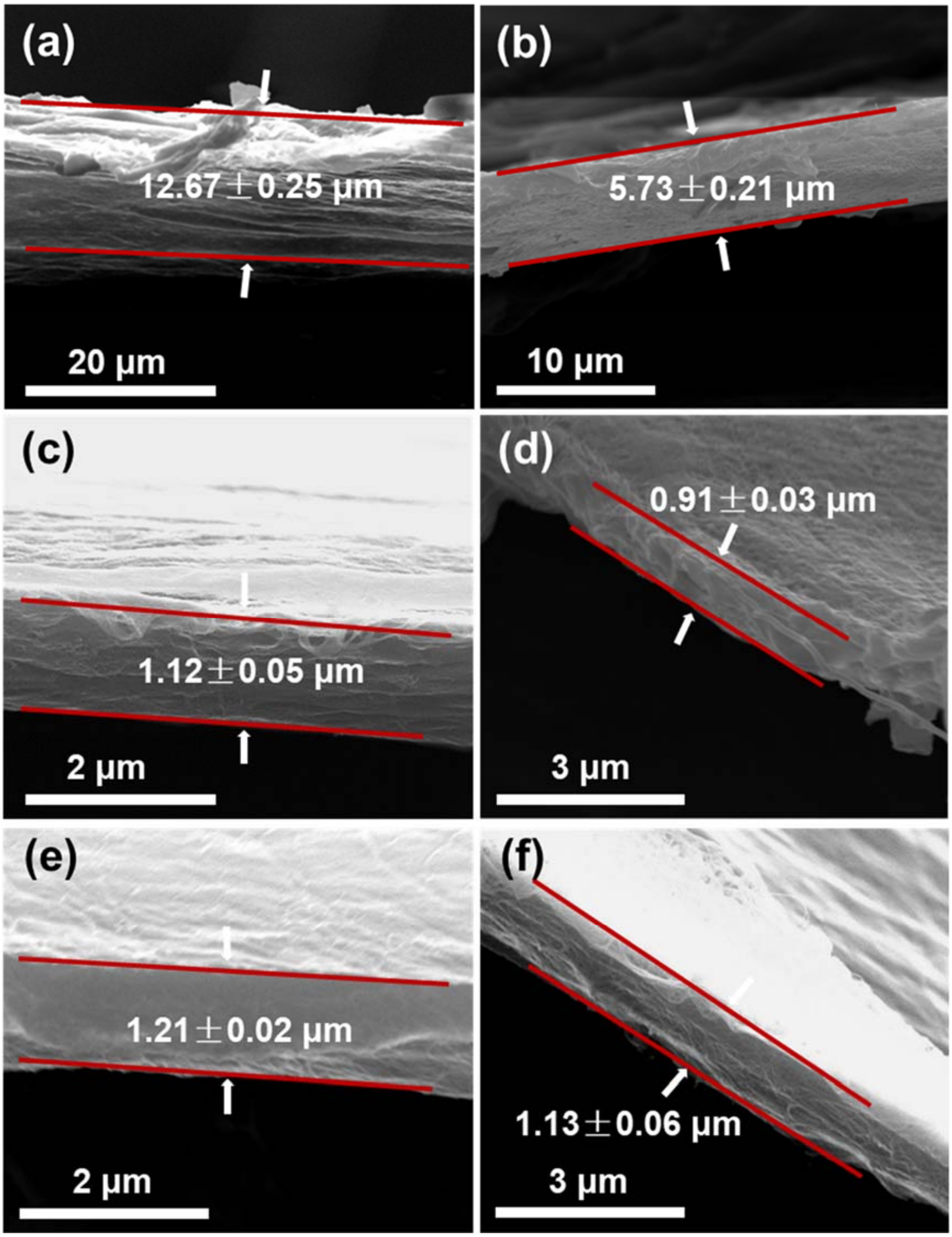

Figure S10. The SEM images of CNT-o films (a), CNT-e films (b), CNT-o/c films (c), CNT-e/c films (d), CNT-o/c-PEI films (e), CNT-e/c-PEI films (f).

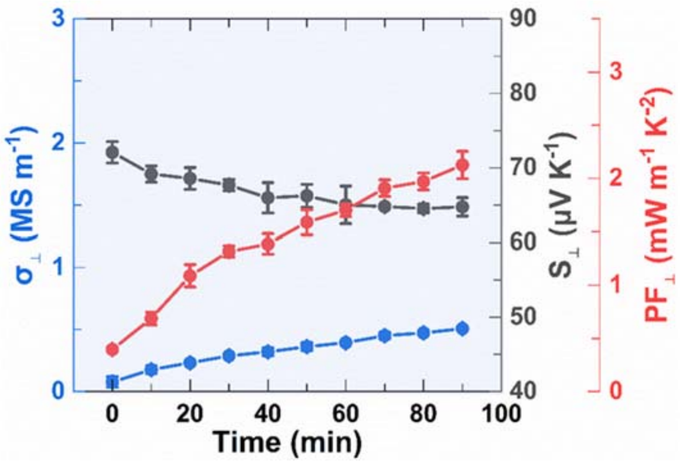

Figure S11. The TE properties in the perpendicular direction of CNT-e film as a function of compressing time.

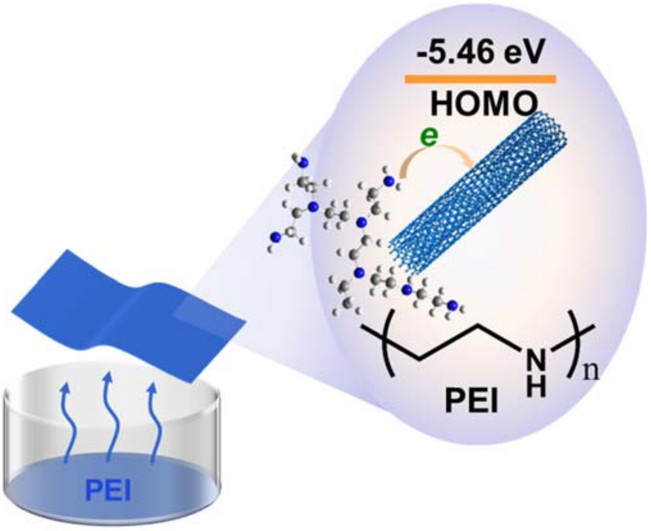

Figure S12. Illustration of vapor doping process.

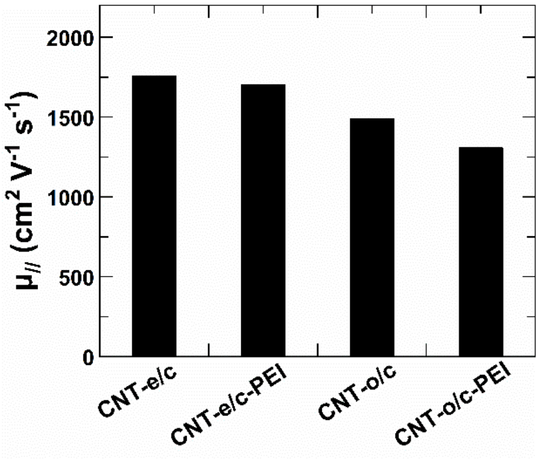

Figure S13. The weight mobility in the parallel direction ($\mu_{//}$) of CNT-o/c films, CNT-e/c films, CNT-o/c-PEI films and CNT-e/c-PEI films.

It is challenging to get the accurate value of carrier mobility for CNT films due to the quantum confinement effect as reported by Tanabe *et al.* in literature.[34, 35] In addition, the contained Fe nanoparticles made the Hall measurement data of the CNT films worse. Therefore, we used weighted mobility reported by Snyder's group to evaluate the variation of carrier mobility in CNT films, since the weighted mobility had a similar trend with the Hall mobility, as reported in the previous literature.[1, 3, 4, 36] The weighted mobility of the obtained matched well with the previously reported literature for CNT.[37]

The weighted mobility of CNT films after PEI treatment exhibited a slight decrease, as shown in Figure S13**.** The slightly reduced weighted mobility can be attributed to the introducing of non-conducting PEI molecules, as suggested in the previous literature.[10, 38]

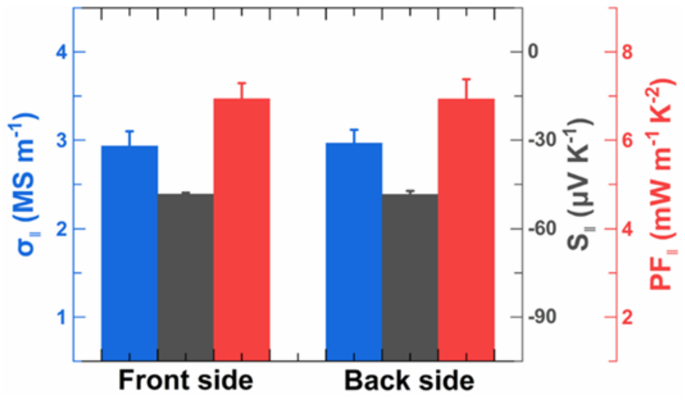

Figure S14. The thermoelectric performance between the front and back surfaces of CNT-e/c-PEI films.

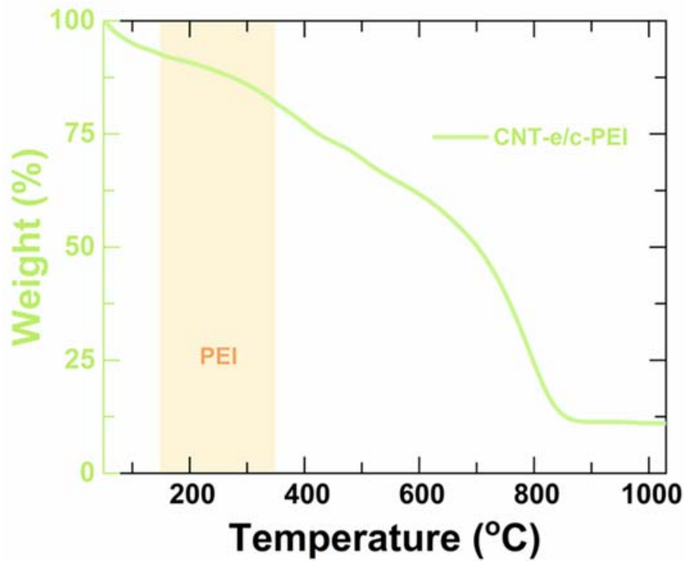

Figure S15. The TGA curve of CNT-e/c films and CNT-e/c-PEI films.

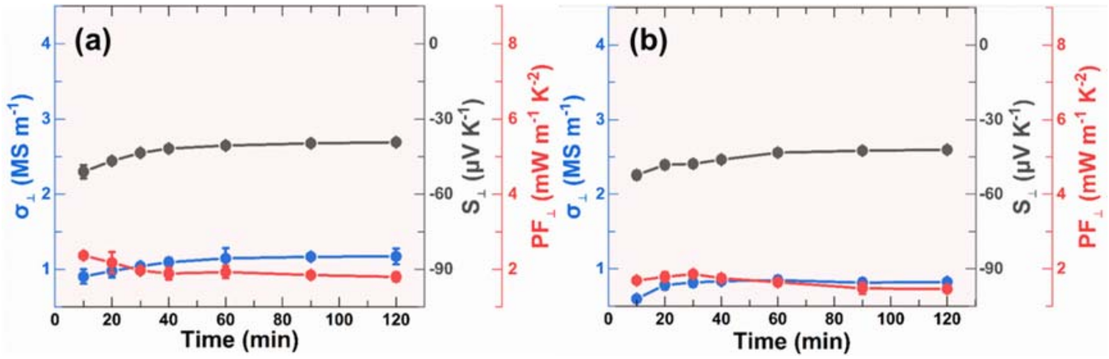

Figure S16. The TE properties in the perpendicular direction of CNT-o/c-PEI films (a) and CNT-e/c-PEI films (b) as a function of vapor treatment time.

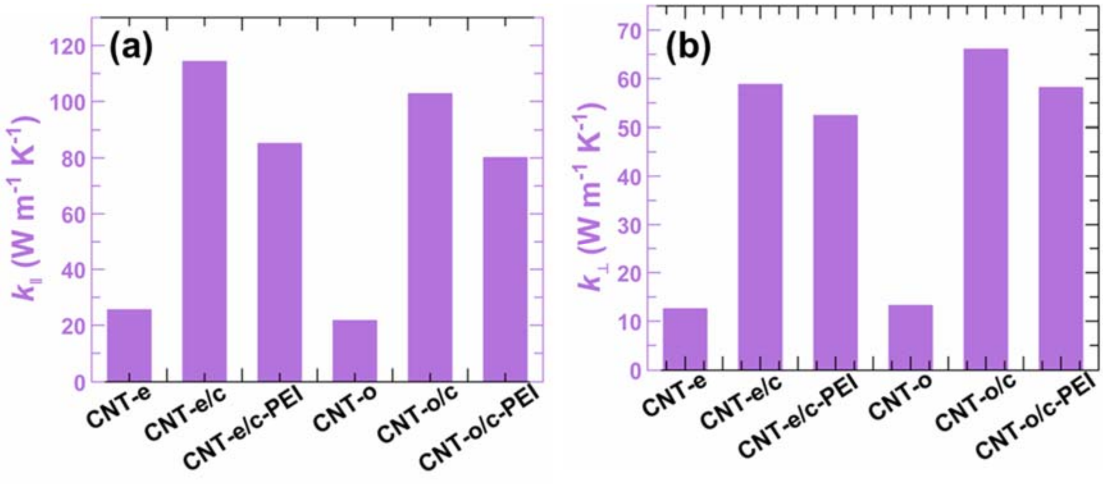

Figure S17. The thermal conductivity of CNT films.

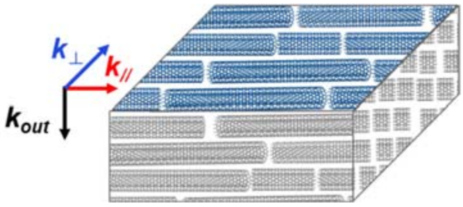

Figure S18. Schematic of thermal conductivity in different directions of carbon nanotube films.

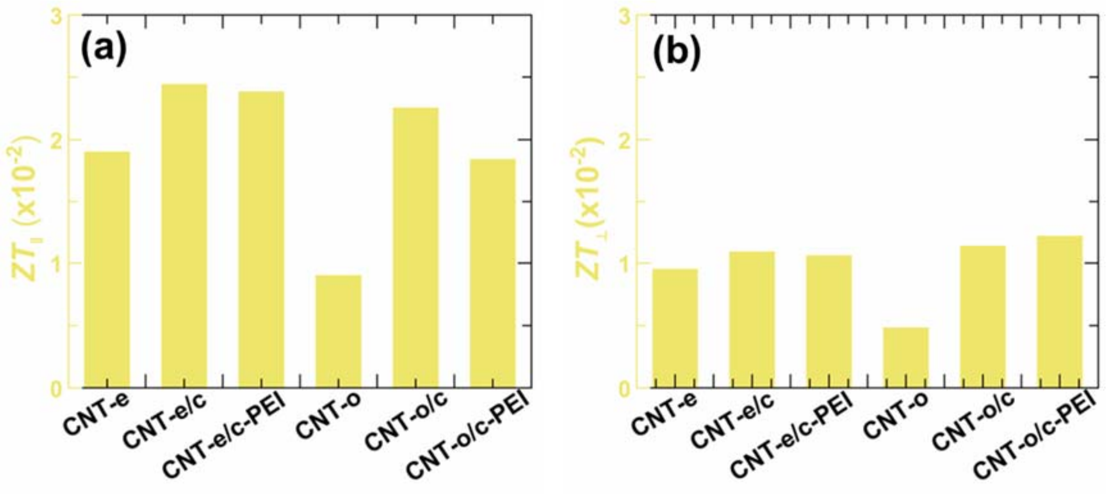

Figure S19. The ZT value of CNT films.

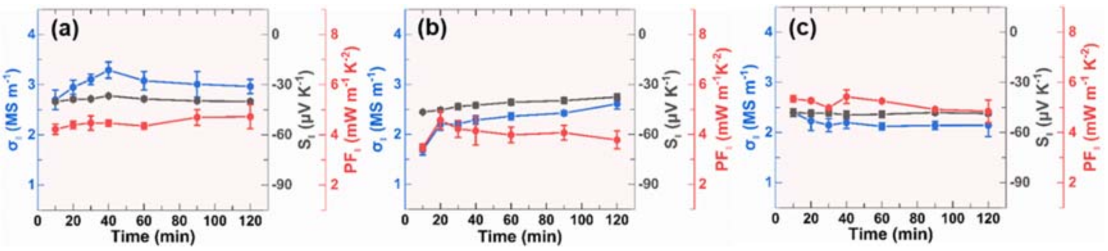

Figure S20. The TE properties in parallel direction of CNT-e film as a function of vapor treatment time for Me-TBD (a), TBD (b) and TMG (c).

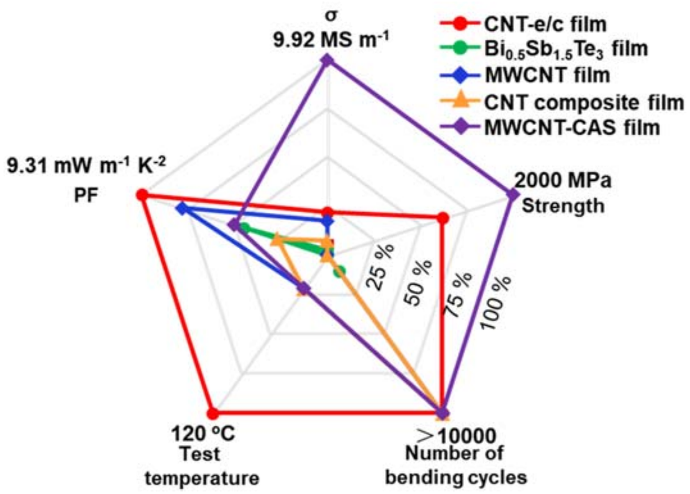

Figure S21. Comparison of comprehensive performance of CNT-e/c films including $\sigma_{\parallel}$, $PF_{\parallel}$ flexibility, mechanical strength and temperature stability.

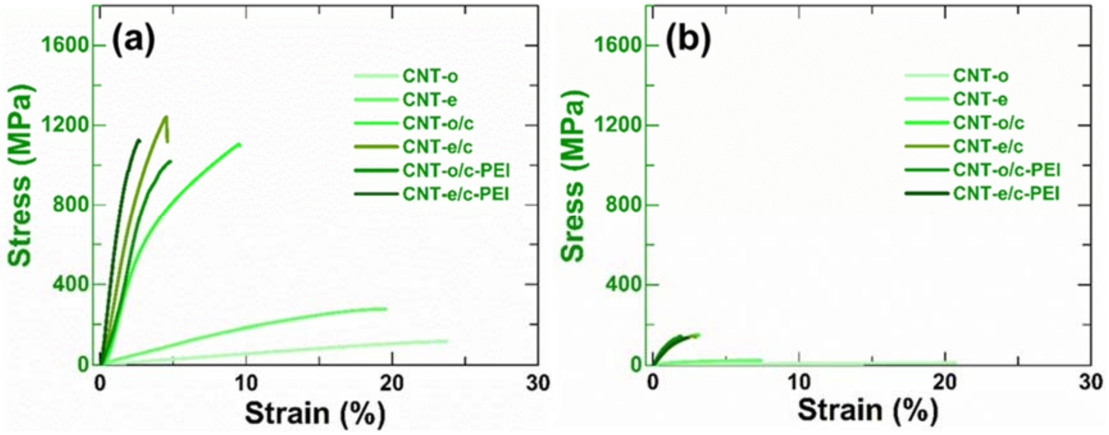

Figure S22. The strain-stress of CNT films in parallel (a) and perpendicular (b) direction.

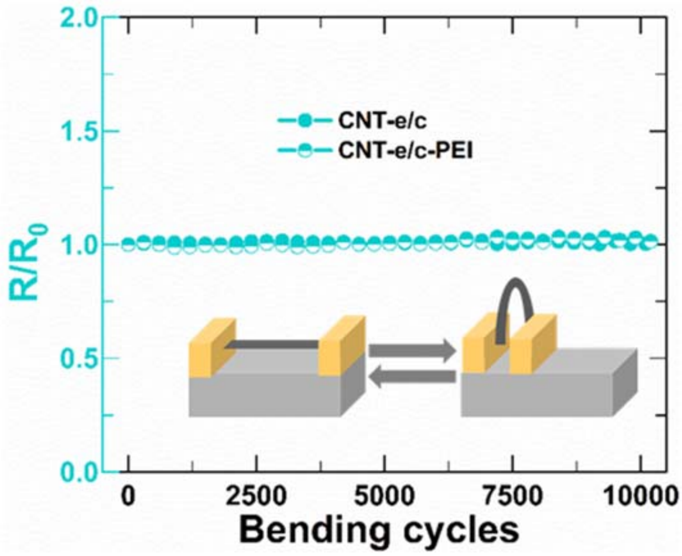

Figure S23. The resistance varied as a function of bending cycles of a strip of CNT-e/c and CNT-e/c- PEI films.

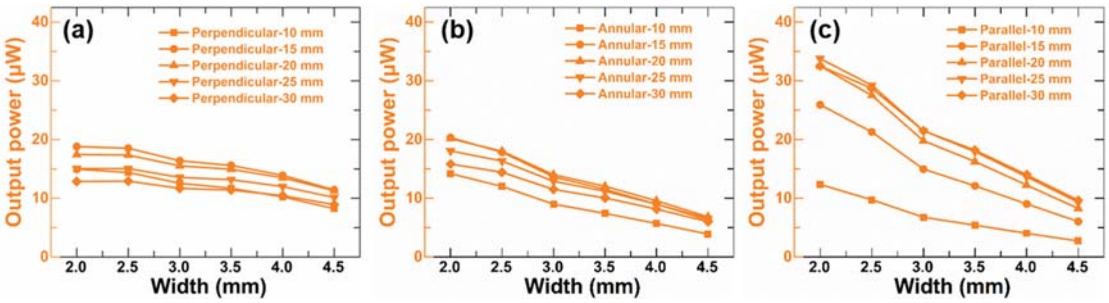

Figure S24. COMSOL simulation results for output power of perpendicular (a), annular (b) and parallel TEG (c) as a function of structural variations with FF=0.5.

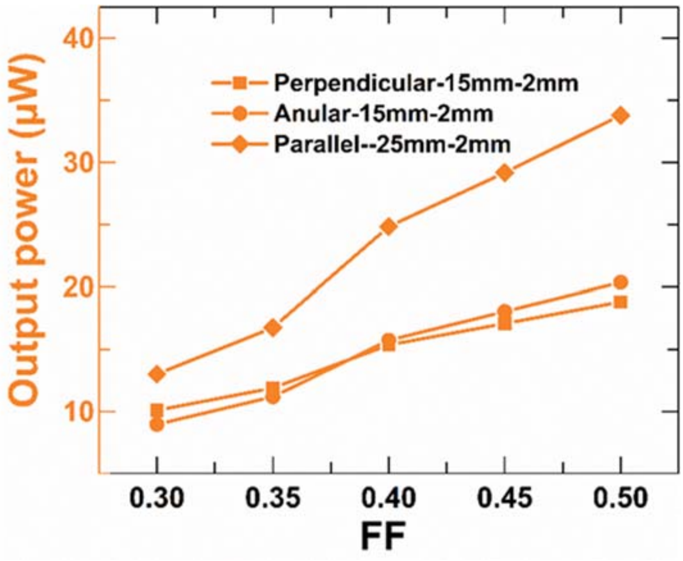

Figure S25. COMSOL simulation results for output power of parallel, perpendicular and annular TEG as a function of FF.

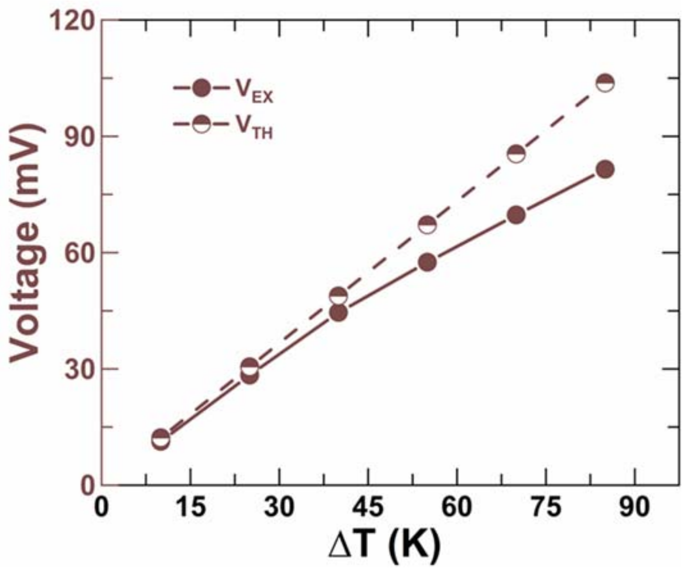

Figure S26. The open-circuit voltage in different temperature differences of SP-TEG/par.

The theoretical open-circuit voltage (V_{TH}) was calculated from the equation of $V_{TH} = N(|S_p| + |S_n|)\Delta T$, where N was the number of p−n modules and S_p and S_n were the Seebeck coefficients of p-type and n-type TE materials, respectively. When the temperature difference was 85 K, the maximum experimental open-circuit voltage (V_{EX}) was 81.5 mV, which was lower than V_{TH} (103 mV, Figure S26).

The theoretical output power (P_{TH}) was calculated from the equation of $P_{TH} = \frac{V_{th}^2}{4R_{in}}$, where R_{in} was the resistance of TEG. When R_{in} was 56 Ω and the temperature difference was 85 K, the P_{TH} was 47.4 μW, which was larger than the experimental output power (29.7 μW) of SP-TEG/par. The output power of SP-TEG/par measured experimentally was only 60.1 % of the theoretical output power. The reason for experimental output power being lower than the theoretical values can be attributed to the fact that the poor heat dissipation at the cold side results in the higher temperature of the cold side compared to the ambient temperature, thus leading to a lower temperature difference between the hot side and the cold side of the TEG. The same issue has also been reported in the previous literature with low experiment to theory values.[39, 40, 41]

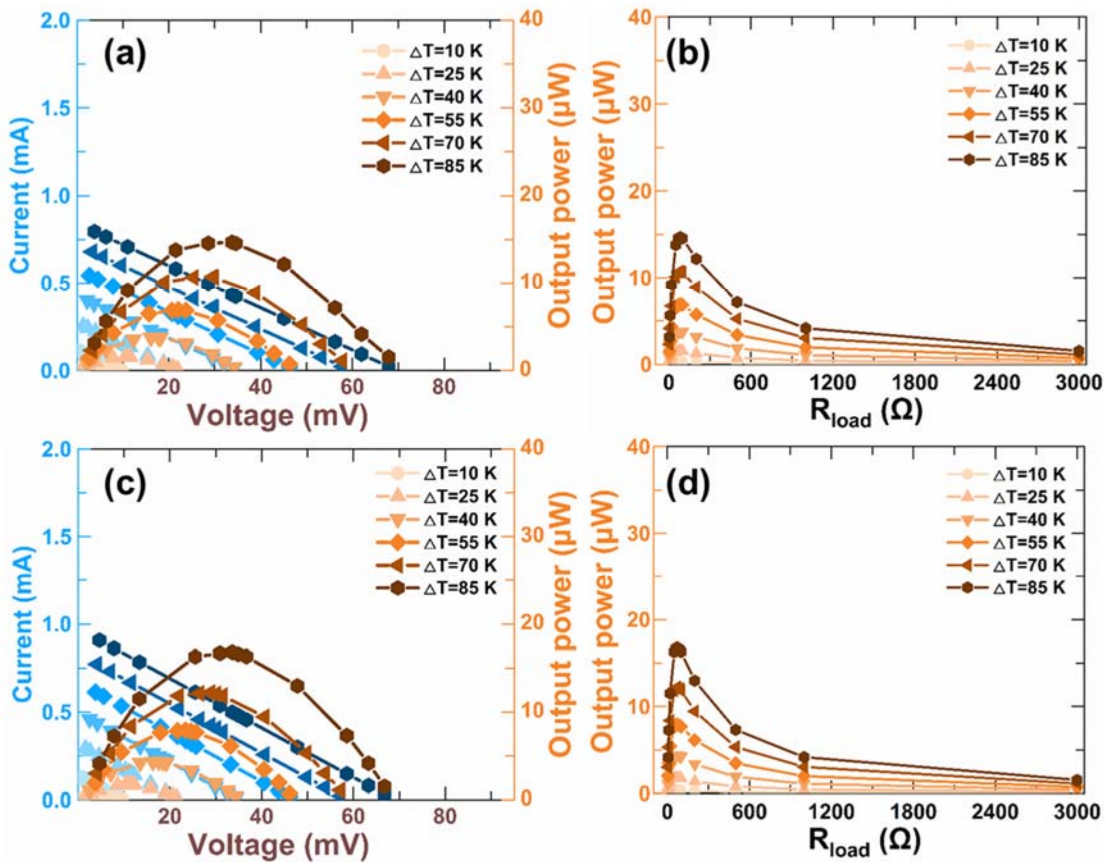

Figure S27. The voltage-current curves of per-TEG (a) and ann-TEG (c) at different temperature differences. The output power of per-TEG (b) and ann-TEG (d) as a function of load resistance.

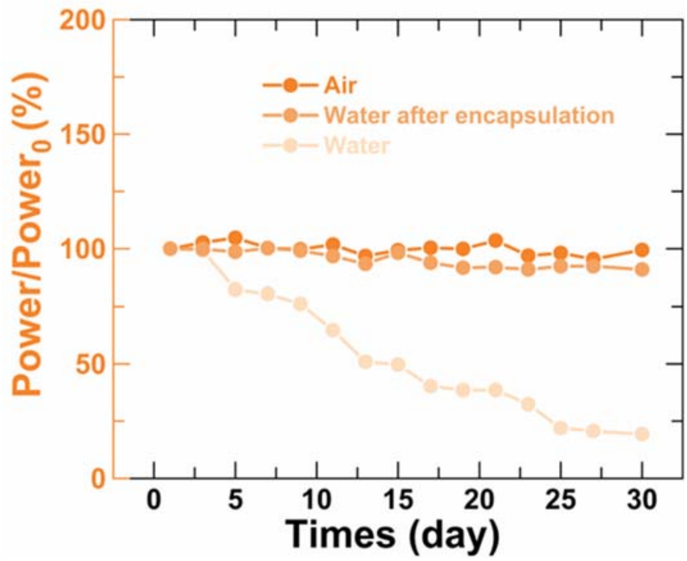

Figure S28. The output power stability of par-TEG (in air, in water and in water after encapsulation).

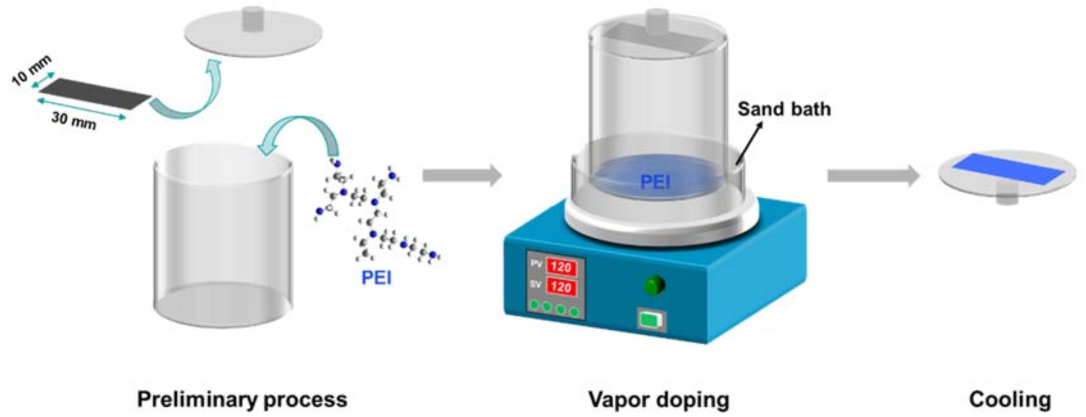

Figure S29. Schematic of the vapor doping process.

Table S1. The sheet resistance of CNT-e/c films.

		σ (MS m^{-1})	Sheet resistance calculated by σ (Ω □$^{-1}$)	Sheet resistance measured by the van der Pauw method (Ω □$^{-1}$)
CNT-e/c film	//	2.17±0.07	0.51±0.03	0.5±0.01

Table S2. The maximum weighted mobility of CNT-o/c-PEI films and CNT-e/c-PEI films.

Materials	$\sigma_{//}$(MS m^{-1})	$S_{//}$ (μV K^{-1})	$\mu_{//}$ (cm^2 V^{-1} s^{-1})
CNT-o/c-PEI-120 min	2.85	-38.14	1307.36
CNT-e/c-PEI-120 min	3.16	-44.65	1703.31

Table S3. The thermal conductivity of CNT-o films, CNT-e films, CNT-o/c films CNT-e/c films, CNT-o/c-PEI films and CNT-e/c-PEI films.

Materials		PF ($mW\ m^{-1}\ K^{-2}$)	k ($W\ m^{-1}\ K^{-1}$)	k_{eff} ($W\ m^{-1}\ K^{-1}$)	ZT
CNT-e	⊥	0.4	12.57	30.57	9.5×10^{-3}
	//	1.7	25.59	102.09	1.9×10^{-2}
CNT-e/c	⊥	2.13	58.81	154.66	1×10^{-2}
	//	9.31	114.3	532.78	2.5×10^{-2}
PEI CNT-e/c	⊥	1.86	52.42	136.12	1.1×10^{-2}
	//	6.75	85.23	388.98	2.4×10^{-2}
CNT-o	⊥	0.21	13.26	22.71	4.8×10^{-3}
	//	0.65	21.73	50.98	9×10^{-3}
CNT-o/c	⊥	2.52	66.09	179.49	1.1×10^{-2}
	//	7.71	102.72	449.74	2.2×10^{-2}
PEI CNT-o/c	⊥	2.37	58.19	164.84	1.2×10^{-2}
	//	4.92	80.18	301.58	1.8×10^{-2}

Table S4. Comparison of calculated effective thermal conductivity κ_{eff} for representative materials with a ΔT of 1 K at 300 K.

Materials		PF (mW m^{-1} K^{-2})	k (W m^{-1} K^{-1})	k_{eff} (W m^{-1} K^{-1})	Ref.
This work	CNT-e/c	9.31	114.3	532.78	
	CNT-e/c-PEI	6.75	85.23	366.48	
CNT-based TE Materials	MWCNT film	7.25	99.4	426	10
	CAS-CNT film	4.66	45.9	256	5
	CNT film	1.8	88.48	170	9
	CNT film	1	62.7	108	9
Metals	Co	15	100	775	42
	$YbAl_3$	16	21	741	43
	$CePd_3$	7.7	10	365	44
	AgPd	5.1	36	266	8
Conventional TE materials	$Cu_{0.9}Bi_{0.1}AgSe$	5.8	4.7	265	45
	Bi_2Te_3	4.5	1.2	203	46
	Bi_2Te_3/CNT composite	4.4	1.69	200	47
	$Mg_3Bi_{1.25}Sb$	4.1	1.5	186	48
	$Bi_2Te_{2.3}Se_{0.7}$	2.8	1.32	127	49

Table S5. Comparison of in-plane ZT values of CNT films at room temperature.

Materials	PF ($mW\ m^{-1}\ K^{-2}$)	k ($W\ m^{-1}\ K^{-1}$)	ZT	Ref.
CNT-e/c film	9.31	114.3	2.5×10^{-2}	This work
CSA-MWCNT film	4.66	45.9	3.0×10^{-2}	5
MWCNT film	7.25	99.4	2.2×10^{-2}	10
MWCNT film	0.34	5.5	1.9×10^{-2}	50
MWCNT film	~1.05	21.9	1.4×10^{-2}	5
SWCNT film	2.2×10^{-5}	9.8	7.0×10^{-3}	51
SWCNT film	$\sim1.1\times10^{-2}$	~1.0	3.3×10^{-3}	52
SWCNT-8022-CN6CP film	0.16	17.2	2.8×10^{-3}	53
Doped SWCNT film	2.3×10^{-5}	39.0	2.0×10^{-3}	51
MWCNT film	-	26.9	$\sim6.5\times10^{-4}$	9

Table S6. Physical properties of the materials used in finite-element analyses.

	Electrical conductivity (MS m^{-1})		Seebeck coefficient (μV K^{-1})	Thermal conductivity (W m^{-1} K^{-1})	
	//	⊥		//	⊥
p-type TE leg	2.17	0.51	64	114.28	58.81
n-type TE leg	2.94	0.82	-47	85.23	52.42

Reference：

1. Liu Y*, et al.* Defect Engineering in Solution-Processed Polycrystalline SnSe Leads to High Thermoelectric Performance. *Acs Nano* **16**, 78-88 (2022).

2. Jin Y*, et al.* Contrasting roles of trivalent dopants M (M = In, Sb, Bi) in enhancing the thermoelectric performance of Ge0.94M0.06Te. *Acta Materialia* **252**, 118926 (2023).

3. Snyder GJ, Snyder AH, Wood M, Gurunathan R, Snyder BH, Niu C. Weighted Mobility. *Adv Mater* **32**, 2001537 (2020).

4. Oanh Kieu Truong L*, et al.* Compensation of Zn substitution and secondary phase controls effective mass and weighted mobility in In and Ga co-doped ZnO material. *J Materiomics* **7**, 742-755 (2021).

5. Wang H*, et al.* Acid enhanced zipping effect to densify MWCNT packing for multifunctional MWCNT films with ultra-high electrical conductivity. *Nat Commun* **14**, 380-380 (2023).

6. Hu Q*, et al.* Double doping approach for unusually stable and large n-type thermoelectric voltage from p-type multi-walled carbon nanotube mats. *J Mater Chem A* **8**, 13095-13105 (2020).

7. Wang Y*, et al.* Mass-produced metallic multiwalled carbon nanotube hybrids exhibiting high N-type thermoelectric performances. *J Mater Chem A* **9**, 3341-3352 (2021).

8. Komatsu N*, et al.* Macroscopic weavable fibers of carbon nanotubes with giant thermoelectric power factor. *Nat Commun* **12**, 4931 (2021).

9. Sun X*, et al.* Anisotropic Electrical Conductivity and Isotropic Seebeck Coefficient Feature Induced High Thermoelectric Power Factor >1800 µW m⁻¹ K⁻² in MWCNT Films. *Adv Funct Mater* **32**, 2203080 (2022).

10. Li K*, et al.* Densification Induced Decoupling of Electrical and Thermal Properties in Free-Standing MWCNT Films for Ultrahigh p- and n-Type Power Factors and Enhanced ZT. *Small* **19**, 2304266 (2023).

11. Dini Y, Faure-Vincent J, Dijon J. How to overcome the electrical conductivity limitation of carbon nanotube yarns drawn from carbon nanotube arrays. *Carbon* **144**, 301-311 (2019).

12. Sheng ZM, Wang JN. Growth of magnetic carbon with a nanoporous and graphitic structure. *Carbon* **47**, 3271-3279 (2009).

13. Jung Y, Kim T, Park CR. Effect of polymer infiltration on structure and properties of carbon nanotube yarns. *Carbon* **88**, 60-69 (2015).

14. Jin Q*, et al.* Flexible layer-structured Bi₂Te₃ thermoelectric on a carbon nanotube scaffold. *Nat Mater* **18**, 62-+ (2019).

15. Xu S*, et al.* Computation-guided design of high-performance flexible thermoelectric modules for sunlight-to-electricity conversion. *Energy Environ Sci* **13**, 3480-3488 (2020).

16. Wang Y*, et al.* Green biopolymer-CNT films exhibit high thermoelectric power factor and electrical conductivity for low temperature heat energy harvesting. *J Mater Chem A* **10**, 25740-25751 (2022).

17. Dai X*, et al.* All-Automated Fabrication of Freestanding and Scalable Photo-Thermoelectric Devices with High Performance. *Adv Mater*, 2312570 (2024).

18. Adachi J, Kurosaki K, Uno M, Yamanaka S. Effect of porosity on thermal and electrical properties of polycrystalline bulk ZrN prepared by spark plasma sintering. *J Alloys Compd* **432**, 7-10 (2007).

19. Goodall R, Weber L, Mortensen A. The electrical conductivity of microcellular metals. *J Appl Phys* **100**, 044912 (2006).

20. Hammoud H, Vaucher S, Valdivieso F. Dielectric and thermal properties of cerium dioxide up to 1000 °C and the effect of the porosity for microwave processing studies. *Thermochim Acta* **617**, 83-89 (2015).

21. Chun K-Y*, et al.* Highly conductive, printable and stretchable composite films of carbon nanotubes and silver. *Nat Nanotechnol* **5**, 853-857 (2010).

22. Hu H*, et al.* Thermoelectric Cu₁₂Sb₄S₁₃-Based Synthetic Minerals with a Sublimation-Derived Porous Network. *Adv Mater* **33**, 2103633 (2021).

23. Suemori K, Uemura S. High thermoelectric performance of post mechanical treated carbon nanotube films with polystyrene binder. *Appl Phys Lett* **116**, 081902 (2020).

24. Wang JN, Luo XG, Wu T, Chen Y. High-strength carbon nanotube fibre-like ribbon with high ductility and high electrical conductivity. *Nat Commun* **5**, 3848 (2014).

25. Ziouche K, Bel-Hadj I, Bougrioua Z. Thermoelectric properties of nanostructured porous-polysilicon thin films. *Nano Energy* **80**, 105553 (2021).

26. Fu M*, et al.* Significant influence of film thickness on the percolation threshold of multiwall carbon nanotube/low density polyethylene composite films. *Appl Phys Lett* **94**, (2009).

27. Loa I. Raman spectroscopy on carbon nanotubes at high pressure. *J Raman Spectrosc* **34**, 611-627 (2003).

28. Chen C, *et al.* Sub-10-nm graphene nanoribbons with atomically smooth edges from squashed carbon nanotubes. *Nat Electron* **4**, 653-663 (2021).

29. Hu M, *et al.* Compressed carbon nanotubes: A family of new multifunctional carbon allotropes. *Sci Rep* **3**, (2013).

30. Mao J, *et al.* Advances in thermoelectrics. *Adv Phys* **67**, 69-147 (2018).

31. Liu B, Hu J, Zhou J, Yang R. Thermoelectric Transport in Nanocomposites. *Materials* **10**, (2017).

32. Wen N, *et al.* Investigations of Morphology and Carrier Transport Characteristics in High-Performance PEDOT:PSS/Tellurium Binary Composite Fibers Produced via Continuous Wet-Spinning. *Adv Funct Mater*, 2315677 (2024).

33. Lin Z, *et al.* The cross-interface energy-filtering effect at organic/inorganic interfaces balances the trade-off between thermopower and conductivity. *Nanoscale* **14**, 9419-9430 (2022).

34. Cancado LG, *et al.* Quantifying Defects in Graphene via Raman Spectroscopy at Different Excitation Energies. *Nano Lett* **11**, 3190-3196 (2011).

35. Altshuler BL, Lee PA. DISORDERED ELECTRONIC SYSTEMS. *Phys Today* **41**, 36-44 (1988).

36. Zhang T, *et al.* Regulation of Ge vacancies through Sm doping resulting in superior thermoelectric performance in GeTe. *J Mater Chem A* **10**, 3698-3709 (2022).

37. Park KT, *et al.* Highly Integrated, Wearable Carbon-Nanotube-Yarn-Based Thermoelectric Generators Achieved by Selective Inkjet-Printed Chemical Doping. *Adv Energy Materi* **12**, 2200256 (2022).

38. Li K, *et al.* Enhanced thermoelectric performance and tunable polarity in 2D Cu2S-phenol superlattices composites for solar energy conversion. *Nano Energy* **84**, 105902 (2021).

39. Zhou Q, *et al.* Leaf-Inspired Flexible Thermoelectric Generators with High Temperature Difference Utilization Ratio and Output Power in Ambient Air. *Adv Sci* **8**, 2004947 (2021).

40. Kim SJ, We JH, Cho BJ. A wearable thermoelectric generator fabricated on a glass fabric. *Energy Environ Sci* **7**, 1959-1965 (2014).

41. Dai X, *et al.* Joint-Free Single-Piece Flexible Thermoelectric Devices with Ultrahigh

Resolution p–n Patterns toward Energy Harvesting and Solid-State Cooling. *ACS Energy Lett* **6**, 4355-4364 (2021).

42. Watzman SJ*, et al.* Magnon-drag thermopower and Nernst coefficient in Fe, Co, and Ni. *Phys Rev B* **94**, 144407 (2016).

43. Rowe DM, Kuznetsov VL, Kuznetsova LA, Min G. Electrical and thermal transport properties of intermediate-valence YbAl₃. *J Phys D Appl Phys* **35**, 2183-2186 (2002).

44. Boona SR, Morelli DT. Enhanced thermoelectric properties of CePd_{3-x}Pt_x. *Appl Phys Lett* **101**, 101909 (2012).

45. Ishiwata S*, et al.* Extremely high electron mobility in a phonon-glass semimetal. *Nat Mater* **12**, 512-517 (2013).

46. Poudel B*, et al.* High-thermoelectric performance of nanostructured bismuth antimony telluride bulk alloys. *Science* **320**, 634-638 (2008).

47. Liu S*, et al.* Thermoelectric Performance Enhancement of Film by Pulse Electric Field and Multi-Nanocomposite Strategy. *Small* **17**, 2100554 (2021).

48. Pan Y*, et al.* Mg₃(Bi,Sb)₂single crystals towards high thermoelectric performance. *Energy Environ Sci* **13**, 1717-1724 (2020).

49. Hu L, Zhu T, Liu X, Zhao X. Point Defect Engineering of High-Performance Bismuth-Telluride-Based Thermoelectric Materials. *Adv Funct Mater* **24**, 5211-5218 (2014).

50. Chatterjee K, Negi A, Kim K, Liu J, Ghosh TK. In-Plane Thermoelectric Properties of Flexible and Room-Temperature-Doped Carbon Nanotube Films. *ACS Appl Energy Mater* **3**, 6929-6936 (2020).

51. Nonoguchi Y*, et al.* Simple Salt-Coordinated n-Type Nanocarbon Materials Stable in Air. *Adv Funct Mater* **26**, 3021-3028 (2016).

52. Zhao W*, et al.* Flexible carbon nanotube papers with improved thermoelectric properties. *Energy Environ Sci* **5**, 5364-5369 (2012).

53. Wang Y*, et al.* Understanding the solvent effects on polarity switching and thermoelectric properties changing of solution-processable n-type single-walled carbon nanotube films. *Nano Energy* **93**, 106804 (2022).
